# ASSOCIATION OF MELANOMA AND INCIDENCE OF PARKINSON’S DISEASE: A COHORT STUDY IN US VETERANS

**DOI:** 10.1093/geroni/igae098.2825

**Published:** 2024-12-31

**Authors:** Gregory Scott, Lee Neilson, Vivek Unni, Moriah Arnold

**Affiliations:** Portland VA Medical Center, Portland, Oregon, United States; Oregon Health & Science University, Portland, Oregon, United States; Oregon Health & Science University, Portland, Oregon, United States; Oregon Health & Science University, Portland, Oregon, United States

## Abstract

Melanoma is associated with subsequent Parkinson’s disease (PD) and identifying the clinicopathologic properties of melanoma driving this association could give insight into PD pathophysiology. We undertook a retrospective cohort study of US military Veterans (n=13,737,081) to determine if malignant and premalignant phenotypes were associated with PD by using propensity score matching 4-to-1 and adjustment for competing risk of death and key confounders. Broad cancer categories were obtained from the Oncology VA registry. Data about melanoma were obtained from clinical and pathology reports using manually validated natural language processing. Risk difference and 95% confidence intervals (CI) were estimated by presence or absence of cancer. For broad cancer categories, we found decreased PD incidence associated with multiple malignancies with some novel associations (e.g. HR = 0.4 [0.2,0.5] for myeloma). In terms of melanoma, BRAF and NRAS mutational status was not associated with incident PD. Atypical/dysplastic nevi was associated with increased PD incidence (HR = 1.4 [1.2,1.7]). Melanoma subtypes in the Oncology registry were associated with PD and included superficial spreading type (HR = 1.8 [1.0, 3.3]) and lentigo maligna (HR = 1.6 [1.2, 2.1]). In people with VA pathology reports, this effect was replicated with greater effect size for superficial spreading type (HR = 10.4 [6.5,16.9]) and lentigo maligna (HR = 6.4 [3.4,11.8]) although adjustment for ascertainment bias is needed. These data show PD is associated with melanoma subtypes and precursor lesions and suggest screening for PD could be appropriate in those with melanocytic lesions once early diagnostic tests are developed.

